# Lymphocytic Colitis With Ileal Extension Following Oyster Consumption: A Case Report

**DOI:** 10.7759/cureus.93190

**Published:** 2025-09-25

**Authors:** Rabia Najeeb, Sri Harsha Kanuri, Vraj J Patel, Ali Z Ansari, Gagandeep S Grewal

**Affiliations:** 1 Department of Internal Medicine, Merit Health Wesley, Hattiesburg, USA; 2 Department of Family Medicine, William Carey University College of Osteopathic Medicine, Hattiesburg, USA

**Keywords:** colonic biopsy, food antigen, intraepithelial lymphocytosis, lymphocytic colitis, microscopic colitis, non-bloody diarrhea, oyster consumption, refractory diarrhea, seafood-related illness, watery diarrhea

## Abstract

Lymphocytic colitis is an uncommon cause of chronic, non-bloody diarrhea characterized histologically by increased intraepithelial lymphocytes within the colonic mucosa, typically with preserved architecture. We present a case of an 84-year-old Caucasian male patient with a history of hypertension, obstructive sleep apnea, benign prostatic hyperplasia, and essential tremor, who developed persistent watery diarrhea, profound fatigue, and unintentional weight loss of 22 pounds within several days after consuming fried oysters at a local restaurant. Initial evaluation at multiple facilities revealed hypotension, hypokalemia, and acute kidney injury, with negative infectious stool studies, unremarkable abdominal imaging, and no evidence of celiac disease. Despite supportive care and antimotility therapy, symptoms persisted, and the patient required repeat hospitalization. A computed tomography (CT) scan demonstrated colonic air-fluid levels consistent with a diarrheal state without inflammatory changes. Colonic biopsies revealed classic lymphocytic colitis, with rare extension into the terminal ileum. The patient experienced significant improvement after initiation of intravenous methylprednisolone, with resolution of vomiting, reduction in stool frequency, normalization of electrolytes, and improvement in renal function, sustained over several weeks of follow-up. This case highlights the diagnostic challenges of lymphocytic colitis, which may present with subacute symptoms that mimic irritable bowel syndrome or chronic infection. The temporal association with recent oyster consumption raises the possibility of a food antigen-mediated trigger, an uncommon but clinically relevant consideration in the pathogenesis of lymphocytic colitis.

## Introduction

Lymphocytic colitis is a histologically defined subtype of microscopic colitis, a chronic inflammatory disorder of the colon that is often underdiagnosed due to its subtle endoscopic appearance; hence the term “microscopic” colitis, as inflammation is typically identified only on histologic examination. Microscopic colitis encompasses two main histologic variants: lymphocytic colitis and collagenous colitis [1]. Both are characterized by chronic watery diarrhea, but they differ in their histopathologic features.

In lymphocytic colitis, the defining feature is a marked increase in intraepithelial lymphocytes within the surface epithelium, typically exceeding 20 lymphocytes per 100 epithelial cells, with preservation of normal crypt architecture and an absence of thickened subepithelial collagen [2]. Epidemiologic data from a population-based study in Olmsted County (Minnesota, United States) demonstrated an overall incidence of microscopic colitis of 8.6 cases per 100,000 between 1985 and 2001, with a significant rise in incidence over time from 1.1 per 100,000 at the beginning of the study to 19.6 per 100,000 by its conclusion. Lymphocytic colitis accounted for 5.5 cases per 100,000, whereas collagenous colitis accounted for 3.1 cases per 100,000. The overall prevalence of microscopic colitis in 2001 was 103.0 per 100,000, with lymphocytic colitis comprising 63.7 per 100,000. Incidence increased with advancing age, and while collagenous colitis demonstrated a marked female predominance, no such sex difference was observed for lymphocytic colitis [3].

The pathogenesis of lymphocytic colitis is multifactorial and remains an area of active investigation. The prevailing hypothesis suggests that the condition arises from an aberrant immune response in genetically susceptible individuals, often triggered by environmental or luminal factors [4]. Genetic predisposition may play a role, with certain human leukocyte antigen (HLA) haplotypes, such as HLA-DQ2 and HLA-DQ8, being more prevalent in affected patients. These genetic factors may alter mucosal immune regulation and impair tolerance to luminal antigens [5]. Environmental triggers are varied and include dietary antigens, infections, medications, and possibly alterations in the gut microbiota [6]. Infectious agents have been repeatedly implicated as potential initiators of disease, with bacterial pathogens such as *Campylobacter jejuni*, *Yersinia enterocolitica*, and *Clostridium difficile* being reported in temporal association with lymphocytic colitis onset [7]. It has been proposed that these infections increase intestinal permeability, allowing antigens to penetrate the epithelial barrier and stimulate mucosal lymphocyte activation [4].

## Case presentation

An 84-year-old Caucasian male patient with a past medical history significant for hypertension, obstructive sleep apnea, benign prostatic hyperplasia, and essential tremor presented to the emergency department with persistent watery diarrhea, nausea, vomiting, and generalized fatigue. The patient was accompanied by his wife, who assisted in providing a detailed account of his illness. He reported that the symptoms began suddenly several weeks prior, within several days of consuming a fried oyster at a local restaurant. He noted that he had previously eaten oysters and other shellfish on multiple occasions without any gastrointestinal or allergic reactions. The diarrhea was described as watery, dark brown in color, non-bloody, and occurring one to three times daily, including nocturnal episodes that required him to wear protective undergarments at night. There was no associated mucus, but he reported occasional urgency and incontinence. He denied abdominal pain but endorsed subjective nocturnal chills, decreased appetite, and progressive weight loss of approximately 22 pounds since symptom onset. The patient also reported profound fatigue, feeling “drained” after minimal activity, and requiring increased rest during the day. He denied any prior history of chronic diarrhea, abdominal pain, or other gastrointestinal complaints before this episode. His family history was negative for food allergies, celiac disease, inflammatory bowel disease, autoimmune thyroid disease, or other autoimmune conditions. His long-term medications included lisinopril, tamsulosin, and primidone, all of which he had been taking for several years without recent changes. He was not on non-steroidal anti-inflammatory drugs (NSAIDs), selective serotonin reuptake inhibitors (SSRIs), or proton pump inhibitors (PPIs).

Prior to this presentation, he had sought medical attention at another facility, where stool studies for *C. difficile* toxins, ova and parasites, and common bacterial pathogens were negative. A computed tomography (CT) scan of the abdomen and pelvis without contrast demonstrated no acute abnormalities, and he was discharged with a presumptive diagnosis of infectious diarrhea, prescribed diphenoxylate-atropine (Lomotil) as needed, and referred to gastroenterology for outpatient evaluation. Despite adherence to supportive measures, his symptoms persisted and became more debilitating, prompting further evaluation.

At the time of arrival to the emergency department, the patient appeared well-developed and appropriately groomed, in no acute respiratory distress, but his overall affect conveyed fatigue and malaise. Vital signs were notable for hypotension, with an initial blood pressure of 80/50 mmHg, which was confirmed on repeat measurement. He was afebrile, with an oral temperature within normal limits, and maintained normal oxygen saturation on room air without signs of increased work of breathing. His pulse rate was slightly bradycardic but regular, and his respiratory rate was within normal limits. Two liters of intravenous lactated Ringer’s solution were administered promptly, resulting in normalization of his blood pressure and improved skin turgor. Cardiopulmonary examination was unremarkable, with clear lung fields bilaterally on auscultation, no adventitious breath sounds, and a regular cardiac rhythm without murmurs, rubs, or gallops. The abdominal examination revealed a soft, non-distended abdomen with normal active bowel sounds in all quadrants. There was no hepatosplenomegaly, no localized or diffuse tenderness to palpation, and no evidence of guarding, rebound tenderness, or palpable masses. The skin was warm and dry, without rashes, jaundice, or petechiae. Neurologic examination demonstrated that the patient was alert and oriented to person, place, and time, with intact cranial nerves II through XII, symmetrical motor strength, normal sensation to light touch and pressure, 2+ deep tendon reflexes, and no focal deficits or cerebellar signs.

Initial laboratory evaluation on admission demonstrated evidence of prerenal acute kidney injury, with elevated serum creatinine of 1.53 mg/dL and blood urea nitrogen of 21 mg/dL. The patient also had significant hypokalemia (3.1 mmol/L), mild hyperchloremia (111 mmol/L), and a low serum bicarbonate level (21 mmol/L), consistent with a mild metabolic acidosis likely due to ongoing gastrointestinal losses and volume depletion. Other electrolytes, including sodium, calcium, magnesium, and phosphate, were within normal limits. Hematologic testing revealed a mild normocytic anemia, with hemoglobin of 13.9 g/dL and hematocrit of 40.1%, while white blood cell and platelet counts remained within normal range. Liver function tests and serum proteins were unremarkable. Inflammatory markers, including C-reactive protein (CRP) and erythrocyte sedimentation rate (ESR), were within normal limits, and extensive infectious stool studies (bacterial culture, *C. difficile* polymerase chain reaction (PCR), ova and parasite examination, and a viral PCR panel) were negative. Specific immunoglobulin E (IgE) testing for shellfish allergens, fecal inflammatory markers (calprotectin and lactoferrin), pancreatic elastase, and a comprehensive nutritional assessment (vitamin B12, folate, and fat-soluble vitamins) were not obtained during the patient’s workup. Following intravenous fluid resuscitation, repeat laboratory studies showed improvement across these parameters, including normalization of potassium to 3.8 mmol/L, reduction of creatinine to 1.1 mg/dL, and correction of acid-base status with bicarbonate rising to 24 mmol/L. Complete details of the patient’s laboratory results, including electrolyte trends, renal function parameters, and hematologic indices, are provided in Table 1.

**Table 1:** Laboratory test results at admission and after intravenous fluid resuscitation, showing correction of electrolyte imbalances and improvement in renal function. IV: intravenous; PCR: polymerase chain reaction

Lab Test	Admission Value	Post-IV Fluid Resuscitation Value	Reference Range
Sodium	143 mmol/L	140 mmol/L	135 – 145 mmol/L
Potassium	3.1 mmol/L	3.8 mmol/L	3.5 – 5.0 mmol/L
Chloride	111 mmol/L	105 mmol/L	98 – 107 mmol/L
Bicarbonate	21 mmol/L	24 mmol/L	22 – 29 mmol/L
Blood urea nitrogen	21 mg/dL	15 mg/dL	7 – 20 mg/dL
Creatinine	1.53 mg/dL	1.1 mg/dL	0.6 – 1.3 mg/dL
Glucose	100 mg/dL	98 mg/dL	70 – 110 mg/dL
Calcium	8.8 mg/dL	8.7 mg/dL	8.6 – 10.2 mg/dL
Magnesium	1.9 mg/dL	1.8 mg/dL	1.7 – 2.2 mg/dL
Phosphate	3.7 mg/dL	3.5 mg/dL	2.5 – 4.5 mg/dL
Hemoglobin	13.9 g/dL	13.5 g/dL	13.5 – 17.5 g/dL
Hematocrit	40.1 %	39.0 %	41 – 53 %
White blood cells	10.9 ×10³/µL	9.5 ×10³/µL	4.0 – 10.5 ×10³/µL
Platelets	227 ×10³/µL	230 ×10³/µL	150 – 450 ×10³/µL
Mean corpuscular volume	90 fL	89 fL	80 – 100 fL
Mean corpuscular hemoglobin	31 pg	30 pg	27 – 33 pg
Mean corpuscular hemoglobin concentration	34 g/dL	33 g/dL	32 – 36 g/dL
Anion gap	11 mmol/L	9 mmol/L	8 – 16 mmol/L
Estimated glomerular filtration rate	50 mL/min/1.73 m²	65 mL/min/1.73 m²	≥ 60 mL/min/1.73 m²
Aspartate aminotransferase	25 U/L	22 U/L	10 – 40 U/L
Alanine aminotransferase	20 U/L	18 U/L	7 – 56 U/L
Alkaline phosphatase	88 U/L	85 U/L	44 – 147 U/L
Total bilirubin	0.8 mg/dL	0.7 mg/dL	0.1 – 1.2 mg/dL
Albumin	3.9 g/dL	3.8 g/dL	3.5 – 5.0 g/dL
Total protein	6.7 g/dL	6.5 g/dL	6.0 – 8.3 g/dL
C-reactive protein	3.9 mg/L	4.1 mg/L	< 10 mg/L
Erythrocyte sedimentation rate	7.6 mm/hr	7.3 mm/hr	< 20 mm/hr
Stool cultures	Negative	—	Negative
Clostridioides difficile PCR	Negative	—	Negative
Ova and parasite exam	Negative	—	Negative
Viral PCR panel	Negative	—	Negative
Celiac serologies	Negative	—	Negative

**Figure 1:**
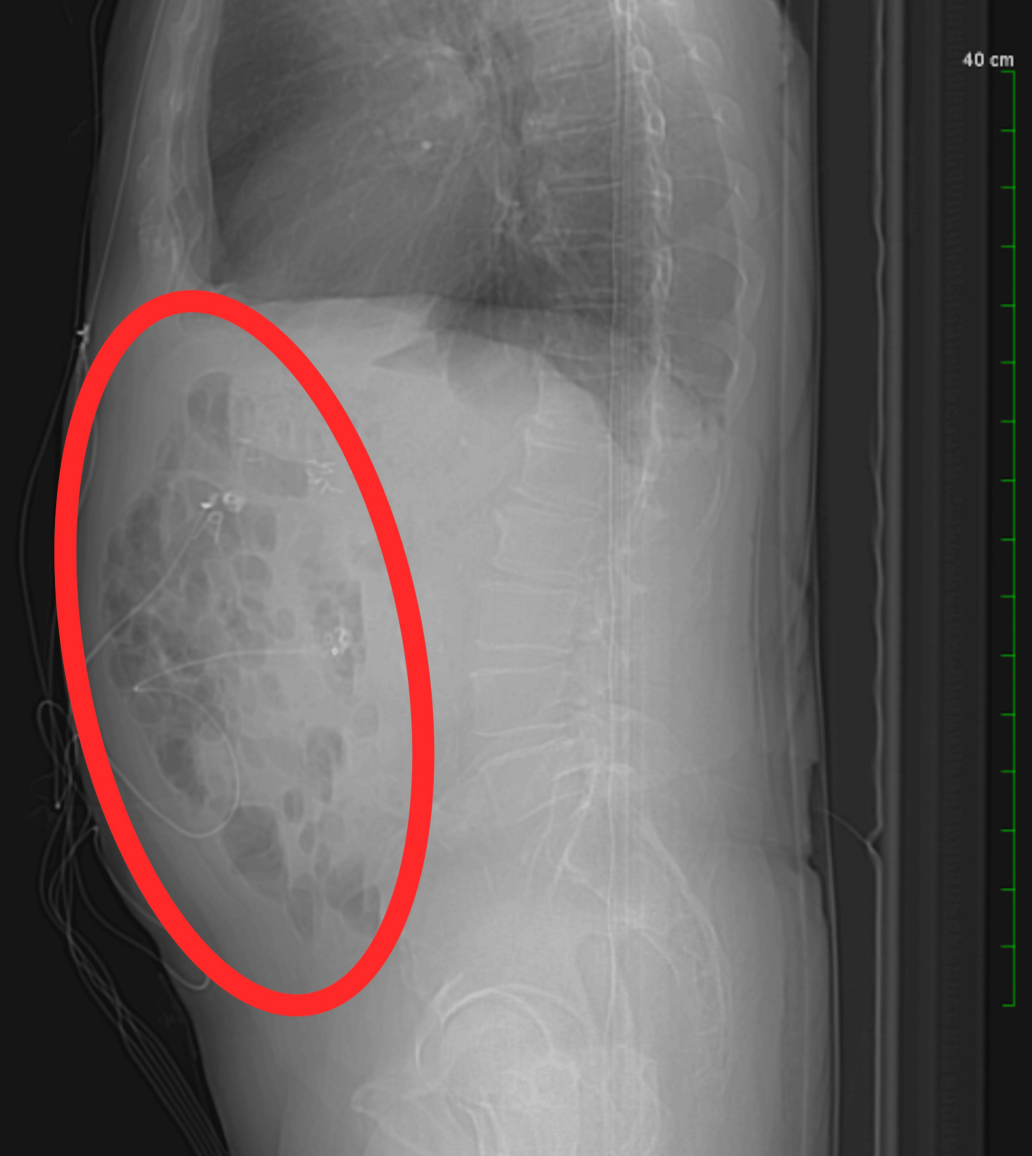
Contrast-enhanced CT scan of the abdomen and pelvis demonstrating multiple air-fluid levels within the colon, consistent with a diarrheal state (red oval). CT: computed tomography

A CT scan of the abdomen and pelvis with contrast performed during this admission demonstrated multiple air-fluid levels within the colon, consistent with a diarrheal state (Figure 1). Additional findings included mild lumbar spondylosis, bilateral renal cortical cysts, chronic changes of bilateral femoral head osteonecrosis, densely calcified coronary atherosclerosis, a dilated post-cholecystectomy common bile duct consistent with reservoir effect, and a small fat-containing left inguinal hernia. Bladder wall thickening was observed, likely secondary to chronic outlet obstruction from prostatomegaly. There was no evidence of bowel obstruction, perforation, or inflammatory mass.

**Figure 2:**
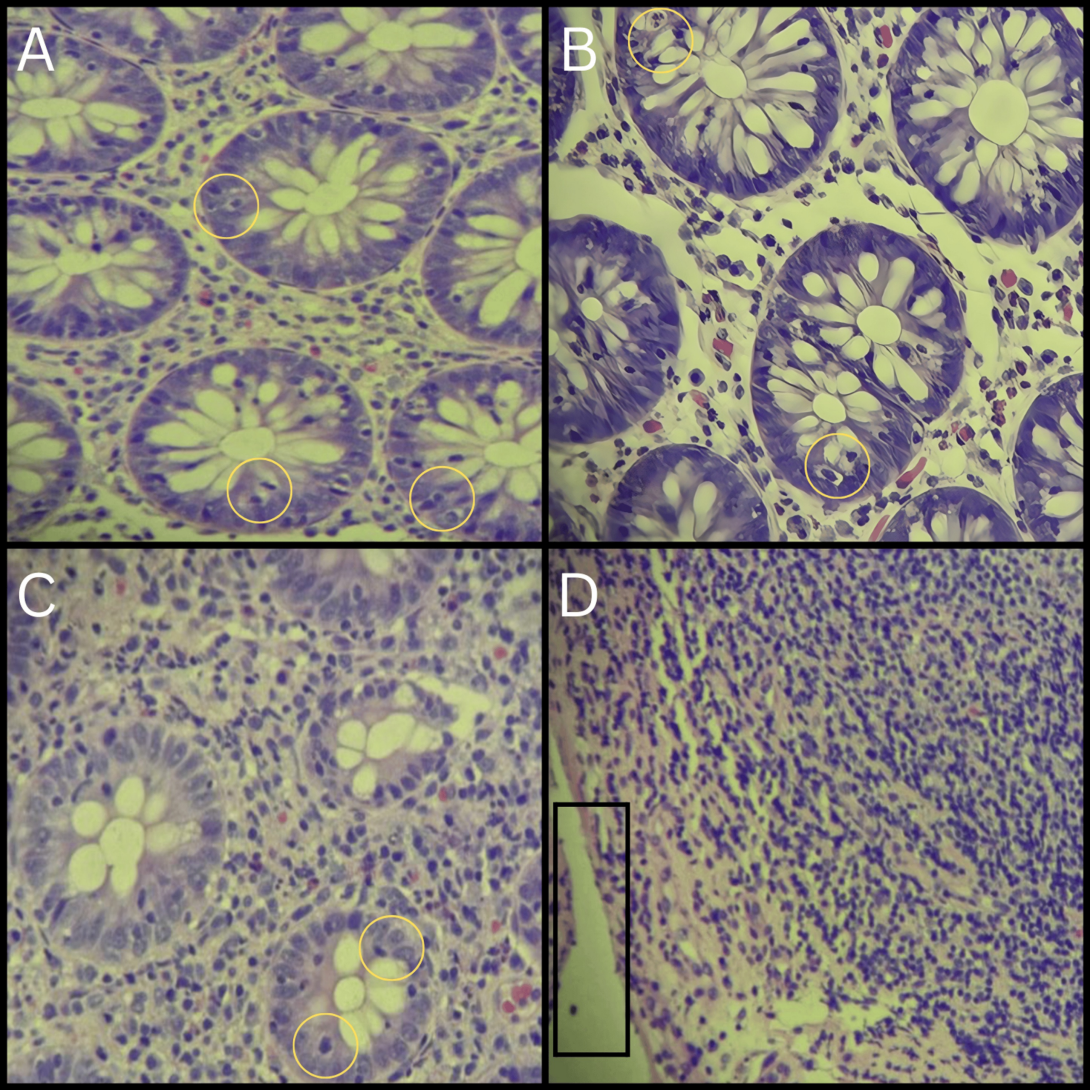
Biopsy images Ascending colon (A), transverse colon (B), and descending colon (C) show cryptitis (yellow circles), with neutrophil infiltration into the crypt epithelium. Terminal ileum (D) shows epithelial lymphocytosis (black rectangle).

Given the chronicity of symptoms, negative infectious workup, and persistent diarrhea despite supportive measures, gastroenterology was consulted for further evaluation. An unprepared colonoscopy was performed, which demonstrated a normal mucosal appearance throughout the colon and terminal ileum, with the exception of a small polyp identified in the ascending colon that was removed during the procedure. Multiple biopsies were obtained from the ascending, transverse, and descending colon, as well as the terminal ileum, to investigate microscopic pathology. Histopathologic examination demonstrated diffuse intraepithelial lymphocytosis, with an average of 25-30 intraepithelial lymphocytes per 100 epithelial cells across all colonic sites sampled, exceeding the diagnostic threshold for lymphocytic colitis. The lamina propria exhibited chronic inflammation with cryptitis and focal crypt abscesses, while crypt architecture remained preserved without basal plasmacytosis. These findings were diagnostic of lymphocytic colitis. The ascending (Figure 2A), transverse (Figure 2B), and descending (Figure 2C) colon biopsies each showed quantitatively similar features, confirming the diffuse involvement. Terminal ileum biopsy (Figure 2D) revealed epithelial lymphocytosis consistent with lymphocytic ileitis, supporting contiguous disease extension. Additionally, one biopsy specimen from the ascending colon contained a tubular adenoma without evidence of high-grade dysplasia, which was completely excised during the colonoscopy.

Following the colonoscopy and histologic confirmation of lymphocytic colitis, the patient was promptly initiated on intravenous methylprednisolone to address the active inflammatory process. Within 48 hours of corticosteroid therapy, he demonstrated marked clinical improvement, characterized by a significant reduction in stool frequency and urgency, with stools becoming more formed and less watery. Accompanying gastrointestinal symptoms, such as nausea and vomiting, resolved completely. This rapid symptomatic relief allowed the patient to gradually resume oral intake, which he tolerated well, negating the need for continued intravenous hydration. Laboratory monitoring revealed normalization of previously noted electrolyte abnormalities, including correction of hypokalemia and chloride levels, and restoration of acid-base balance. Renal function improved, with serum creatinine and blood urea nitrogen returning to baseline values, reflecting resolution of the prerenal acute kidney injury. After clinical stabilization, the patient was transitioned to oral prednisone at a dose of 40 mg daily, with a planned tapering regimen tailored to minimize potential corticosteroid-related adverse effects while maintaining disease control.

At discharge, the patient was advised to maintain adequate hydration and adhere strictly to the oral prednisone regimen. Detailed counseling was provided regarding the importance of recognizing early signs of dehydration, recurrent or worsening diarrhea, and any new or severe gastrointestinal symptoms, which would warrant prompt medical evaluation. Additionally, he was educated on monitoring for urinary retention, given his history of benign prostatic hyperplasia and observed bladder wall thickening on imaging, and instructed to seek urgent care if such symptoms developed. At follow-up visits over the next six weeks, the patient demonstrated marked clinical improvement with resolution of diarrhea, normalization of bowel habits, and recovery of appetite and energy. No symptom recurrence was documented during this period, and he reported avoidance of further oyster or shellfish consumption. Treatment response was assessed clinically through resolution of diarrhea and weight stabilization, though no formal scoring tool was applied.

## Discussion

This case represents an uncommon and clinically significant presentation of lymphocytic colitis involving diffuse colonic inflammation with histologic extension into the terminal ileum, a finding rarely reported in the literature. Traditionally, lymphocytic colitis is defined by its confinement to the colon, with the small intestine remaining histologically unremarkable [2]. The presence of ileal involvement in this patient suggests either a contiguous extension of the inflammatory process or a more generalized mucosal immune activation, possibly indicating that lymphocytic colitis lies along a broader spectrum of mucosal inflammatory diseases. Such findings invite reconsideration of whether lymphocytic colitis is strictly a colonic disorder or, in certain contexts, part of a pan-intestinal immune response triggered by environmental, dietary, or infectious factors. Although we cannot prove that oysters caused the illness, the timing strongly suggests they may have acted as a trigger in a genetically or immunologically susceptible individual. This “two-hit” scenario, in which a triggering event superimposes upon a primed immune background, aligns with the proposed multifactorial pathogenesis of microscopic colitis [4].

The risk factors implicated in lymphocytic colitis can be broadly divided into three interrelated categories: priming factors, triggering factors, and drug-induced contributors [8]. Priming factors create a baseline susceptibility in the host and include genetic predispositions, particularly certain HLA haplotypes such as HLA-DR3, DQ1, DQ2, and DQ3, which have been repeatedly associated with lymphocytic colitis and other immune-mediated gastrointestinal disorders. This genetic landscape is often accompanied by coexisting autoimmune diseases, most notably celiac disease, autoimmune thyroiditis, and type 1 diabetes mellitus [5]. In addition, immunosuppression, whether due to hematologic or solid malignancies, post-solid organ transplantation immunosuppressive therapy, chemotherapy, or prior radiation exposure, further compromises the mucosal barrier and alters immune surveillance [9]. Collectively, these priming factors appear to weaken epithelial tight junction integrity, disrupt mucosal homeostasis, and diminish the gut’s capacity to appropriately regulate immune tolerance, thereby rendering it more vulnerable to subsequent inflammatory insults.

Triggering factors are those that actively initiate disease activity in a primed individual. These include infectious agents such as *C. jejuni*, *C. difficile*, and *Y. enterocolitica*, each of which can transiently disrupt the epithelial barrier, increase paracellular permeability, and introduce bacterial antigens into the lamina propria [7]. In addition, dietary antigens, particularly gluten in genetically predisposed individuals, and certain environmental exposures can act as persistent luminal irritants. These antigens and microbial components can exhibit molecular mimicry with host structures, thereby confusing the immune system into mounting an autoimmune-like response [10]. This aberrant adaptive immune activation involves both the T cell-mediated arm, driven by CD4+ helper T cells and cytotoxic CD8+ T cells, and the B cell-mediated arm, leading to the production of autoantibodies and pro-inflammatory cytokines such as tumor necrosis factor alpha (TNF-α), interferon gamma (IFN-γ), and interleukin-1 beta (IL-1β), as well as chemokines that recruit additional immune effector cells [11]. Over time, this creates a sustained inflammatory environment in the colonic mucosa.

Drug-induced lymphocytic colitis represents another clinically important category, with numerous medications implicated in either initiating or perpetuating the inflammatory cascade. Commonly reported culprits include nonsteroidal anti-inflammatory drugs, selective serotonin reuptake inhibitors, proton pump inhibitors, and certain antihypertensive agents [12]. The mechanisms are multifactorial, involving both direct mucosal irritation and alteration of gut microbiota composition, which can amplify immune dysregulation in susceptible individuals.

At the tissue level, the inflammatory response in lymphocytic colitis is characterized by a marked accumulation of CD3+ and CD8+ intraepithelial lymphocytes within the surface epithelium, as well as an increased density of lymphocytes and plasma cells in the lamina propria [11]. These intraepithelial lymphocytes interact closely with enterochromaffin cells, specialized neuroendocrine cells of the intestinal epithelium, stimulating excessive serotonin release. Serotonin, in turn, diffuses to adjacent enterocytes and activates nitric oxide synthase pathways, resulting in elevated nitric oxide production. This biochemical interplay produces a dual effect: nitric oxide and serotonin synergistically enhance intestinal motility while simultaneously increasing active secretion of water and electrolytes into the lumen. As a result, stool water content rises significantly, manifesting clinically as persistent secretory diarrhea. The intestine attempts to counterbalance this secretory state through increased production of peptide YY, an inhibitory gut hormone that slows intestinal transit and reduces fluid loss. However, in lymphocytic colitis, this compensatory response is insufficient to overcome the heightened secretory drive and rapid transit time induced by the ongoing inflammatory and neuroendocrine signals. The end result is the hallmark presentation of lymphocytic colitis: chronic, non-bloody, watery diarrhea often accompanied by urgency and nocturnal symptoms, reflecting the persistent imbalance between intestinal secretion and absorption [13].

The definitive diagnosis of lymphocytic colitis hinges on histopathological examination of colonic biopsies, which characteristically demonstrate an increased density of intraepithelial lymphocytes exceeding 20 per 100 surface epithelial cells. This finding is typically accompanied by a mixed inflammatory infiltrate within the lamina propria composed of lymphocytes, eosinophils, and plasma cells, yet importantly without disruption of the overall mucosal architecture [2]. In the present case, biopsies obtained from multiple colonic segments as well as the terminal ileum revealed classical histological hallmarks of lymphocytic colitis, including chronic inflammation in the lamina propria, cryptitis, and focal crypt abscess formation, while preserving crypt architecture and showing no evidence of basal plasmacytosis. These features were instrumental in differentiating lymphocytic colitis from inflammatory bowel disease, which is characterized by basal lympho-plasmacytosis, crypt distortion, and mucosal architectural abnormalities that were notably absent in this patient [14]. Although chromogranin-A immunostaining was not performed in this case, it is recognized as a highly sensitive and specific adjunct diagnostic tool that can assist in distinguishing lymphocytic colitis from inflammatory bowel disease, given its ability to highlight neuroendocrine components frequently present in lymphocytic colitis [15].

Initial management typically includes supportive care with antidiarrheals such as loperamide, bismuth subsalicylate, and bile acid sequestrants like cholestyramine. For patients with persistent or severe symptoms, corticosteroids are the mainstay of therapy. Budesonide, due to its topical effect and limited systemic absorption, is the preferred agent and has shown efficacy in clinical trials. However, in acute or severe presentations where oral intake is compromised, systemic corticosteroids such as intravenous methylprednisolone may be required for rapid symptom control [12]. This patient responded well to intravenous steroids with significant improvement in stool frequency and consistency, resolution of nausea and vomiting, and normalization of electrolyte abnormalities and renal function. Transitioning to oral prednisone with a planned taper was appropriate to reduce relapse risk while minimizing corticosteroid-associated adverse effects. For steroid-resistant or dependent cases, immunomodulators like azathioprine and methotrexate offer steroid-sparing alternatives. Although rare, surgical interventions such as subtotal colectomy with ileostomy may be necessary for refractory disease to reduce morbidity and mortality [16].

Although most cases of lymphocytic colitis resolve spontaneously or respond well to medical therapy, long-term follow-up remains crucial due to the potential risk of progression to inflammatory bowel disease or colorectal neoplasia. Chronic mucosal inflammation is a well-established risk factor for neoplastic transformation in the gastrointestinal tract, and the incidental finding of a tubular adenoma in this patient highlights the necessity of thorough endoscopic evaluation and vigilant surveillance protocols [17]. These considerations also demonstrate the importance of including lymphocytic colitis in the differential diagnosis of chronic, non-bloody diarrhea, particularly among elderly patients with predisposing risk factors. Moreover, it emphasizes the value of obtaining comprehensive biopsies from both colonic and, when clinically indicated, ileal sites, even in the absence of visible endoscopic abnormalities.

The differential diagnosis for chronic watery diarrhea is broad and includes inflammatory bowel diseases such as ulcerative colitis and Crohn’s disease, infectious colitis, drug-induced colitis, eosinophilic gastroenteritis, and functional disorders like diarrhea-predominant irritable bowel syndrome. Inflammatory bowel disease is typically distinguished by basal plasmacytosis, crypt architectural distortion, and, in Crohn’s disease, transmural inflammation with granulomas, none of which were observed in this patient [14]. Infectious colitis was excluded based on negative stool cultures, ova and parasite exams, and viral PCR testing, as well as the absence of neutrophil-predominant mucosal injury. Drug-induced microscopic colitis, often triggered by NSAIDs, SSRIs, or PPIs, was unlikely given the patient’s medication history. Eosinophilic gastroenteritis, though rare, presents with prominent eosinophilic infiltration rather than lymphocytosis. Functional disorders, while clinically similar, lack histologic inflammation. Together, these considerations highlight the essential role of histopathology in establishing a definitive diagnosis and guiding appropriate management. The interplay of genetic susceptibility, immune dysregulation, microbial triggers, and medication effects continues to be an area of active investigation, with future research aimed at elucidating molecular and immunologic mechanisms that could enhance diagnostic accuracy and foster development of targeted, individualized therapies for lymphocytic colitis.

## Conclusions

Lymphocytic colitis is an important and frequently underdiagnosed cause of chronic, non-bloody diarrhea, particularly in older adults, which can lead to significant complications such as weight loss, electrolyte imbalances, and acute kidney injury if not promptly recognized and treated. This case illustrates the potential for extensive involvement of both the colon and terminal ileum, expanding the clinical and histopathological spectrum of the disease. It emphasizes the necessity of obtaining multiple random biopsies even in the presence of normal-appearing mucosa during endoscopy to establish an accurate diagnosis.

Early and appropriate intervention with corticosteroids, especially in severe cases, can achieve rapid symptomatic relief and restore physiological balance, minimizing morbidity. Additionally, given the variable clinical course and the rare risk of progression to inflammatory bowel disease or colorectal neoplasia, long-term surveillance and individualized patient follow-up are crucial. Heightened clinical suspicion, comprehensive diagnostic evaluation, and timely management of lymphocytic colitis are essential to improving patient outcomes and reducing the burden of this often overlooked gastrointestinal disorder. The temporal association with oyster ingestion is hypothesis-generating and highlights a potential food antigen trigger, though causality cannot be established from a single case.
